# FENDRR Affects COAD Biological Behavior by Inhibiting the DUSP4/CREB/PRKACB Pathway

**DOI:** 10.1155/ijog/2765511

**Published:** 2025-07-01

**Authors:** Hao Zhang, Li Hong, Zirui Zhuang, Qiange Zhang, Feng Zhang, Ruipeng Wang, Jinjing Xu, Youyuan Tang, Xingpo Guo, Ling Gao

**Affiliations:** Department of General Surgery, The First Affiliated Hospital of Soochow University, Suzhou, Jiangsu Province, China

**Keywords:** colon adenocarcinoma, CREB, DUSP4, FENDRR, PRKACB

## Abstract

**Background:** Colorectal cancer (CRC) is acknowledged as the third leading cause of cancer-related mortality, attributed to its high incidence and fatality rates. Long noncoding RNAs (lncRNAs) have emerged as novel biomarkers for the treatment of colon adenocarcinoma. This study is aimed at investigating the function and underlying mechanisms of the lncRNA fetal-lethal noncoding developmental regulatory RNA (FENDRR) in regulating the malignant phenotype of CRC (COAD) cells.

**Methods:** This investigation examined FENDRR expression patterns and their association with clinical outcomes in 496 COAD and 173 READ patients from The Cancer Genome Atlas (TCGA) dataset. Additionally, 10 clinical COAD specimens were collected to validate FENDRR expression levels. Using lentiviral-mediated gene delivery, we stably upregulated FENDRR in HCT-116 cells, with transcriptional changes quantified via qPCR. The tumor biological behavior was evaluated using in vitro experiments, including CCK-8, colony formation, wound healing, transwell assays, and immunofluorescence staining. Protein-level alterations were subsequently confirmed by Western blot.

**Results:** Through bioinformatics evaluation, a notable decrease in FENDRR expression levels was observed in both COAD and READ tissues, with a pronounced link between FENDRR expression and tumor T stage classification in COAD cases. Patients exhibiting diminished FENDRR expression showed worse clinical outcomes in COAD. Enrichment analysis demonstrated significant associations between FENDRR and various signaling cascades, particularly the cAMP pathway. Additionally, immune cell infiltration analysis showed a significant association with FENDRR expression levels. In vitro experiments confirmed that FENDRR overexpression hindered the proliferation, migration, and invasion of cells. Mechanistically, FENDRR has been demonstrated to induce the sinking of the DUSP4/CREB/PRKACB signaling pathway and reverse the epithelial–mesenchymal transition (EMT) pathway, thereby inhibiting tumor growth.

**Conclusion:** We establish FENDRR as a tumor-suppressing gene that plays a significant role in suppressing the advancement and metastatic spread of COAD. These findings underscore its diagnostic and prognostic utility in COAD.

## 1. Introduction

Colorectal cancer (CRC) ranks as the third highest in global incidence rates, representing approximately 10% of all cancer cases and serving as the second leading cause of death from malignant cancers. Although CRC incidence has declined in recent years, the 5-year survival rate remains unsatisfactory, primarily because most patients are diagnosed at advanced metastatic stages [1]. The clinical management of CRC is further complicated by the challenge of early diagnosis and treatment. There is an urgent need to identify novel biomarkers for early screening and prognosis to improve CRC management.

Long noncoding RNAs (lncRNAs) are predominantly localized in the nucleus and cytoplasm and are produced through alternative splicing. lncRNAs are involved in various biological processes, thereby playing a critical regulatory role in the development and progression of malignant tumors [2]. Fetal-lethal noncoding developmental regulatory RNA (FENDRR, also known as FOXF1-AS1) is a novel lncRNA located on chr3q13.31 with four exons and 3099 nucleotides [3]. Research has consistently documented significant reductions in FENDRR expression across various malignancies, including those originating from the colon, lung, bile duct, cervix, prostate, breast, renal, esophageal, gastric, and endometrial tissues. Furthermore, FENDRR has been shown to exhibit tumor suppressor functions, influencing numerous processes such as cell proliferation, programmed cell death, migration, invasion, chemotherapy resistance, and stem cell characteristics. This is achieved through diverse mechanisms, including the sequestration of oncogenic microRNAs and participation in protein interactions [4]. Collectively, these findings indicate that FENDRR exerts a pivotal function in the progression of cancer. Nevertheless, the precise upstream and downstream regulatory pathways through which FENDRR operates remain incompletely understood, necessitating more comprehensive investigation. Additionally, research has revealed strong associations between FENDRR expression and clinicopathological characteristics. For instance, reduced FENDRR levels correlate with higher-stage CRC, while its downregulation is linked to aggressive metastatic behavior in lung cancer. In gastric cancer, FENDRR expression significantly correlates with tumor progression, depth of invasion, and lymph node involvement [5–7]. These insights could contribute to future clinical applications, such as improving diagnostic criteria, prognostic assessment, and therapeutic strategies for various malignancies.

As a key phosphatase within its family, dual-specificity phosphatase 4 (DUSP4), also known as MKP2, is located at the chr8p11.12 locus [8]. These phosphatases are “dual-specificity” because they can dephosphorylate both serine/threonine and tyrosine residues in the cells. Structurally, DUSP4 features the conserved MKPpTXpTY motif, along with three key regions: an N-terminal MKB domain (residues 1–192), a central catalytic DUSP domain (193–336), and a C-terminal segment (337–394) [9]. DUSP4 expression has been observed to be elevated in human tumors, including CRC and KRAS-mutant rectal cancer. Furthermore, increased DUSP4 expression levels in CRC demonstrate a significant association with distant metastasis and poor clinical outcomes, suggesting the oncogenic activity of DUSP4 in CRC. Conversely, reduced DUSP4 levels correlate with aggressive gastric cancer progression, including higher tumor grade, metastatic spread, and diminished patient survival [10]. Consequently, the impact of DUSP4 on diverse tumor types is not uniform. As a critical regulator, the MAPK cascade governs essential cellular functions such as growth, specialization, programmed cell death, and viability. By dephosphorylating ERK, P38, and JNK, DUSP4 inactivates MAPK signaling, modulating key processes like senescence, apoptosis under stress, and malignant transformation [11–16]. Furthermore, DUSP4 has been documented as a factor that influences the progression of CRC by modulating the cAMP signaling pathway. This mechanism is characterized by the involvement of proteins such as cAMP response element–binding protein (CREB) and PRKACA, a subject that is also the focal point of this article [17].

CREB is a nuclear protein consisting of 341 amino acids and is a member of the leucine zipper family of transcription factors. It has been demonstrated to bind to the cAMP response element (CRE) sequence TGACGTCA or to the conserved half CRE sequence TGACG. Its initial identification was in studies related to the promoter of the growth inhibitor gene [18]. The basic leucine zipper (bZIP) structural domain of CREB is responsible for its binding and dimerization to DNA, while the kinase-inducible structural domain (KID) of CREB, which contains nine serine residues, can be phosphorylated and activated by different kinases, thus regulating gene transcription. Upon activation, CREB attracts transcriptional coactivators including CREB-binding protein (CBP), leading to the formation of the CREB-CBP transcriptional complex. The CREB-CBP complex has been demonstrated to initiate CREB-dependent gene transcription [19, 20]. Emerging research reveals upregulated CREB levels across multiple malignancies, such as liver, kidney, ovarian, prostate, lung, gastric, esophageal, pancreatic, and breast cancers. This is closely related to tumor-related progression factors (e.g., proliferation, differentiation, cell cycle regulation, apoptosis, angiogenesis, metastasis, immune surveillance and metabolism, and tumor stem cell generation) [21–24]. However, studies related to CREB in CRC with FENDRR remain to be conducted.

Protein kinase A (PKA) is considered to be indispensable in the cAMP signaling pathway, and its activation modulates numerous critical cellular functions including chromatin remodeling, proliferation, metabolic regulation, differentiation, and cycle control. The main part of its role is played by its catalytic subunit, PRKACB. Upon binding to the two regulatory subunits of the inactive PKA, cAMP separates the regulatory subunit from the catalytic subunit, thereby releasing and activating the catalytic subunit, PRKACB [25]. The activated PKA catalytic subunit has the capacity to phosphorylate certain proteins (e.g., Ser-133 of CREB) within the cell, thereby regulating the activity of these proteins. Consequently, this modulates the expression of associated genes, thereby mediating their biological functions [26]. Research has demonstrated that activated PKA is implicated in the regulation of long-term memory formation [27], and PRKACB has been observed to promote CRC development through the process of CREB phosphorylation. However, no study has yet reported the relationship between PRKACB and FENDRR in CRC.

The epithelial–mesenchymal transition (EMT) is a biological mechanism whereby cells of epithelial origin shed their defining characteristics and adopt traits typical of mesenchymal cells, playing crucial roles in embryogenesis, wound healing, and metastatic cancer spread. Key signaling pathways, including TGF-*β*, Wnt/*β*-catenin, Notch, and NF-*κ*B, tightly modulate this phenomenon. Studies indicate that EMT facilitates migratory and invasive behaviors across diverse malignancies. Indeed, it is widely considered to be the single most important step in the progression of malignancy. This is characterized mainly by a decrease in the adhesive capacity of the cells, remodeling of the cytoskeleton, and an increase in cellular mobility [28]. It has been reported that in bladder cancer, FENDRR can inhibit the EMT process by targeting the miR-18a-5p/AFF4 signaling pathway [29]. However, it has not been investigated whether FENDRR can influence the EMT process in COAD.

This study analyzed the link between FENDRR expression patterns and patient prognosis in COAD. Through in vitro experiments, we confirmed that FENDRR overexpression suppresses colon cancer cell proliferation, migratory capacity, and invasive potential. We have proposed and validated scientific hypotheses concerning the potential mechanisms by which FENDRR affects the malignant characteristics of COAD. These hypotheses were formulated on the basis of previous studies and combined with bioinformatics analysis. Furthermore, we explored the relationship between FENDRR and EMT-related proteins. The findings of this study suggest that FENDRR is a promising diagnostic and prognostic marker for COAD.

## 2. Materials and Methods

### 2.1. Specimen Collection

This investigation analyzed tissue specimens from 10 individuals with confirmed colon carcinoma, all of whom underwent resection procedures at the First Affiliated Hospital of Soochow University from January to December 2024. Notably, preoperative neoadjuvant treatment was not administered to any participant, and histopathological examination postsurgery verified colon adenocarcinoma in all cases. After obtaining written consent from participants and their relatives, both malignant tumor tissues and adjacent normal mucosal samples were harvested. The samples were then utilized to evaluate the expression levels of FENDRR, which were stored in a −80°C refrigerator immediately after ex vivo. The study protocol received ethical approval, and written consent was secured from every participant.

### 2.2. Public Databases and Data Analysis

The GEPIA database (http://gepia.cancer-pku.cn/) was used to get FENDRR expression data. Expression data from The Cancer Genome Atlas (TCGA) (https://portal.gdc.cancer.gov/) database focused on extraction of COAD and READ transcriptome sequencing and clinical information of the corresponding samples, such as age, sex, TNM stage, and survival information. RNA-seq data and clinical information for 455 COAD and 165 READ patients were downloaded from TCGA (https://portal.gdc.cancer.gov/). Based on median FENDRR expression, COAD/READ patients were stratified into high- and low-expression cohorts, with clinical variables categorized for analysis [30]. Chi-square tests assessed univariate associations of FENDRR with clinicopathological features, whereas binary logistic regression evaluated multivariate relationships. We examined FENDRR's association with overall survival (OS) in COAD/READ patients, generating Kaplan–Meier curves via Survival package in R software (Version 4.4.2). Further, univariate and multivariate Cox regression analyses confirmed FENDRR's prognostic significance in COAD, with results visualized as forest plots and nomogram.

### 2.3. Gene Enrichment Analysis

The Limma package provided in the R software (Version 4.4.2) was used to investigate differential expression of RNA. To screen for differential expression of RNA between FENDRR high -and low-expression groups, the threshold was set to “*p* < 0.05 and Log2 (fold change) > 1 or Log2 (fold change) < −1” to generate a differential expression gene volcano plot. For a deeper understanding of the oncogenic role of FENDRR, the ClusterProfiler package in R was used to elucidate and visualize possible gene ontologies (GOs (CC, BP, and MF)) and Kyoto Encyclopedia of Genes and Genomes (KEGG) enrichment analysis, as well as gene set enrichment analysis (GSEA) [31].

### 2.4. Tumor Immunity Estimation Analysis

To assess FENDRR's link to the immune microenvironment in COAD, we compared immune cell infiltration (22 subsets) between high- and low-expression groups using CIBERSORT in R. These differences were visualized via ggplot2 to contrast immune infiltration patterns across FENDRR expression levels.

### 2.5. Cell Culture and Transfection

We obtained HCT-116 cell line from the Shanghai Cell Bank, Chinese Academy of Sciences (Shanghai, China). The HCT-116 cells were cultured in Dulbecco's modified Eagle medium (DMEM, HyClone, United States), supplemented with 1% penicillin–streptomycin (HyClone) and 10% fetal bovine serum (FBS, Excellbio, United States). The cells were maintained under conditions of 37°C and 5% CO_2_ for subsequent analyses. Cells are regularly refreshed with medium and passaged at a ratio of 1:2–1:4, with cell density adjusted to an appropriate level prior to experiments. Transfection experiments are performed in six-well plates when cell density reaches approximately 70%–80% using lentivirus designed and constructed by GenePharma following the manufacturer's protocol. Cellular status was monitored 8–12 h following transfection, followed by replacement of the culture medium with fresh DMEM complete medium at 24 h posttransfection to eliminate residual viral particles. We then screen for successfully transfected cells by adding an appropriate amount of puromycin hydrochloride to the medium. Quantitative PCR (qPCR) is used to assess transfection efficiency.

### 2.6. RT-qPCR

RNA extraction from cellular and tissue samples was conducted using Trizol (Invitrogen, California, United States). The RNA was reverse transcribed into cDNA using a cDNA Synthesis Supermix Kit (Takara, Japan). Subsequently, the expression levels of the target genes were determined by SYBR Premix Ex Taq II (Takara, Japan) on the ABI 7500 fast real-time PCR system (Applied Biosystems, United States). The sequences of the FENDRR primers were forward 5⁣′-CTCCCGTGGAAGCCATTTCT-3⁣′ and reverse 5⁣′-CCTCTGGCTGCGTTTTTCAC-3⁣′. The sequences of the GAPDH primers were forward 5⁣′-TGTGAGGGAGATGCTCAGTG-3⁣′ and reverse 5⁣′-TGTTCCTACCCCCAATGTGT-3⁣′. Gene expression levels were comparatively calculated via the 2^–*ΔΔ*Ct^ approach.

### 2.7. CCK-8 Assay

The assessment of colon cancer cell proliferation was conducted utilizing the CCK-8 assays. A total of 1000 tumor cells were seeded into each well of a 96-well plate. Following a 24-h incubation period, the medium was replaced, and each well received 10 *μ*L of CCK-8 solution. After an additional 2 h of incubation at 37°C, absorbance was measured at 450 nm. These measurements enabled analysis of cellular growth patterns and viability across predetermined time points. This process was repeated continuously for 5 days.

### 2.8. Colony Formation Assay

The cells were transferred to six-well plates and allowed to incubate for a duration of 2 weeks. Following incubation, methanol fixation was performed prior to the application of 0.1% crystal violet stain. Observations and cell counting were performed using an Olympus microscope.

### 2.9. Ki-67 Immunofluorescence (IF) Staining

To test whether FENDRR can alter tumor cell proliferation, we performed IF staining of nuclear Ki-67. Five-micrometer-thick slides were permeabilized with 0.1% Triton-X100 solution for 5 min, washed with PBS, and blocked with 1% bovine serum albumin (Sigma-Aldrich) for 30 min at room temperature. Afterward, the samples were incubated with anti-Ki-67 antibody (Proteintech, No. 27309-1-AP, 1:100) for 1 h at room temperature. The process is continued with three washes with PBS and incubation with secondary antibody for 45 min. Next, slides were washed with PBS and restained with 1 *μ*g/mL DAPI solution.

### 2.10. Wound Healing Assay

A confluent monolayer of colon cancer cells was established in six-well culture plates. Using sterile 200-*μ*L pipette tips, linear scratches were created in the cellular monolayer to generate defined wound areas. Following wound creation, PBS washes removed dislodged cells, and serum-free DMEM was introduced to eliminate potential serum-induced migration effects. Images were taken under a microscope at 0 and 24 h.

### 2.11. Transwell Assay

Transwell chambers (Corning Inc.) were employed to assess cellular migration and invasion capacities, with Matrigel-coated and uncoated variants. Briefly, colon cancer cells that had been transfected (2 × 10^4^) were introduced into 100 *μ*L of serum-free medium (Gibco, Thermo Fisher Scientific Inc.) and placed in the upper chamber. Meanwhile, 500 *μ*L of DMEM supplemented with 10% serum (Shanghai ExCell Biology Inc.) was added to the lower chamber. The upper chamber was incubated for 24 h at 37°C in a 5% CO_2_ atmosphere, followed by fixation of the cells with 4% paraformaldehyde (Beyotime Institute of Biotechnology) for 10 min at room temperature. Afterward, the cells were stained with 0.2%–0.5% crystal violet (Sigma-Aldrich, Merck KGaA) for 10 min at room temperature and subsequently examined under an inverted optical microscope (Shanghai Optical Instrument Factory) for statistical analysis. The migration assay followed a method akin to the invasion assay, with the only distinction being the omission of Matrigel.

### 2.12. Western Blotting

Cells were subjected to protein isolation utilizing RIPA buffer (P0013B, Beyotime, China). Protein quantification was conducted using a BCA protein detection kit (A55864, Thermo Fisher Scientific, United States). Following this, proteins underwent separation via SDS-PAGE and were subsequently transferred to PVDF membranes. These membranes were incubated for 2 h with 5% nonfat milk to minimize nonspecific binding, before undergoing overnight incubation with primary antibodies. The application of secondary antibodies occurred at room temperature for 1 h. Color development was then carried out using an enhanced chemiluminescence (ECL) kit (Millipore, United States) and analyzed with ImageJ software.

## 3. Results

### 3.1. FENDRR Expression Level

**Figure 1:**
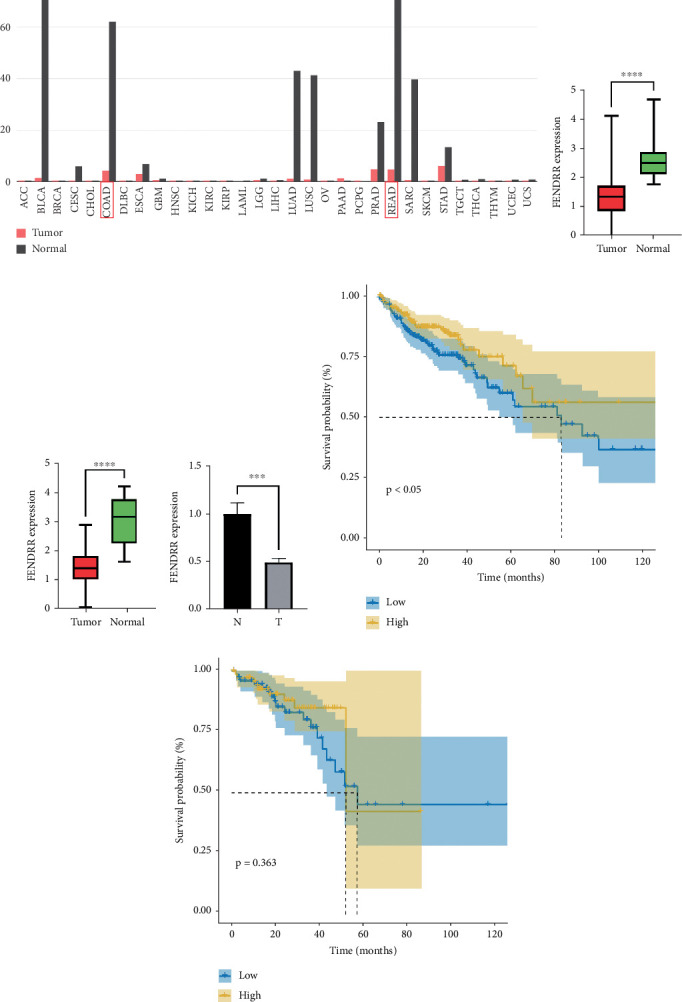
Expression levels of FENDRR and survival analysis. (a) Expression profile of FENDRR across multiple cancer types in the GEPIA database. (b) FENDRR expression in colon adenocarcinoma (COAD) from TCGA database. (c) FENDRR expression in rectum adenocarcinoma (READ) from TCGA database. (d) FENDRR expression in clinical COAD tissue specimens. (e) Kaplan–Meier survival analysis of COAD patients stratified by FENDRR expression. (f) Kaplan–Meier survival analysis of READ patients stratified by FENDRR expression.

As demonstrated in Figure 1a, the GEPIA online database indicated that FENDRR expression was downregulated in bladder urothelial carcinoma, cervical squamous cell carcinoma, endocervical adenocarcinoma, and colon adenocarcinoma, compared to normal tissues, esophageal carcinoma, lung adenocarcinoma, lung squamous carcinoma, prostate adenocarcinoma, rectal adenocarcinoma, and gastric adenocarcinoma (*p* < 0.05). We focused on extracting the mRNA expression of COAD and READ from TCGA database (Figure 1b,c). This finding was further validated through qPCR analysis of clinical specimens, demonstrating significant FENDRR downregulation in COAD tissues (Figure 1d).

### 3.2. Correlation Between FENDRR Expression and Clinicopathological Features of COAD/READ

Using TCGA data, we examined associations between FENDRR levels and clinicopathological features in COAD/READ cases. Univariate chi-square testing (Table S1) revealed that among COAD patients, T stage distribution varied significantly when comparing high versus low FENDRR expression cohorts (*p* < 0.05). However, rectal adenocarcinoma samples demonstrated no statistically meaningful correlations with any clinical parameters (*p* > 0.05). These findings were subsequently confirmed through multivariate logistic regression modeling, which maintained the same pattern of statistical significance (Table S2).

### 3.3. Association of FENDRR Expression With Prognosis in COAD/READ

**Figure 2:**
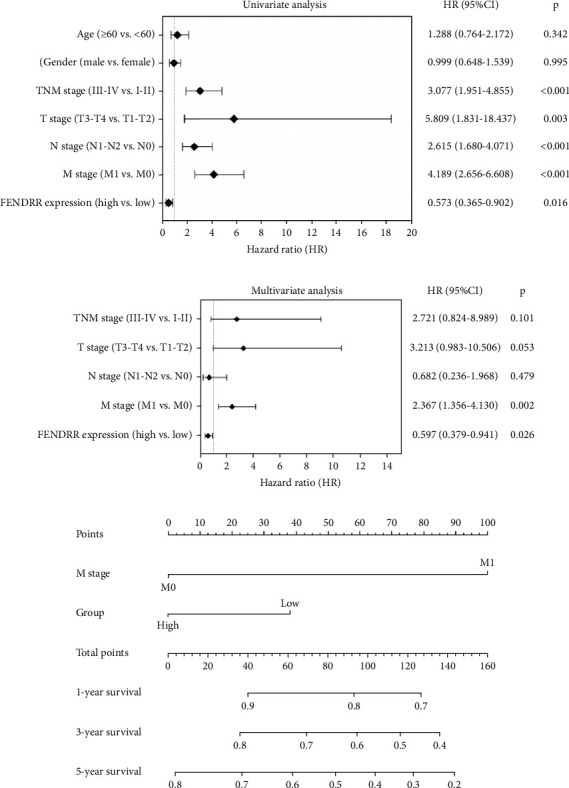
Cox regression analysis and nomogram. (a) Univariate Cox regression analysis. (b) Multivariate Cox regression analysis. (c) Nomogram constructed based on the multivariate regression analysis results.

Figure 1e,f presents Kaplan–Meier analyses illustrating the relationship between FENDRR expression and patient survival outcomes in COAD/READ. Notably, COAD cases exhibiting reduced FENDRR expression showed significantly worse OS rates (*p* < 0.05, Figure 1e). In contrast, READ patients displayed no significant survival differences based on FENDRR expression levels (*p* = 0.363, Figure 1f). Consequently, the subsequent study was focused exclusively on COAD, while READ was excluded. Univariate Cox proportional hazards modeling identified TNM stage, T stage, N stage, and M stage as independent predictors of poor prognosis in COAD, whereas elevated FENDRR expression emerged as a protective factor (Figure 2a). Subsequently, the five independent variables of TNM stage, T stage, N stage, M stage, and FENDRR expression level were incorporated into a multivariate Cox regression model (Figure 2b). This analysis revealed M stage as an independent predictor of adverse outcomes in COAD, whereas elevated FENDRR expression maintained its protective effect. Finally, we further constructed column line graphs based on M stage and FENDRR expression levels, thereby predicting 1-, 3-, and 5-year survival (Figure 2c).

### 3.4. Differential Expression Analysis and Functional Enrichment Analysis

**Figure 3:**
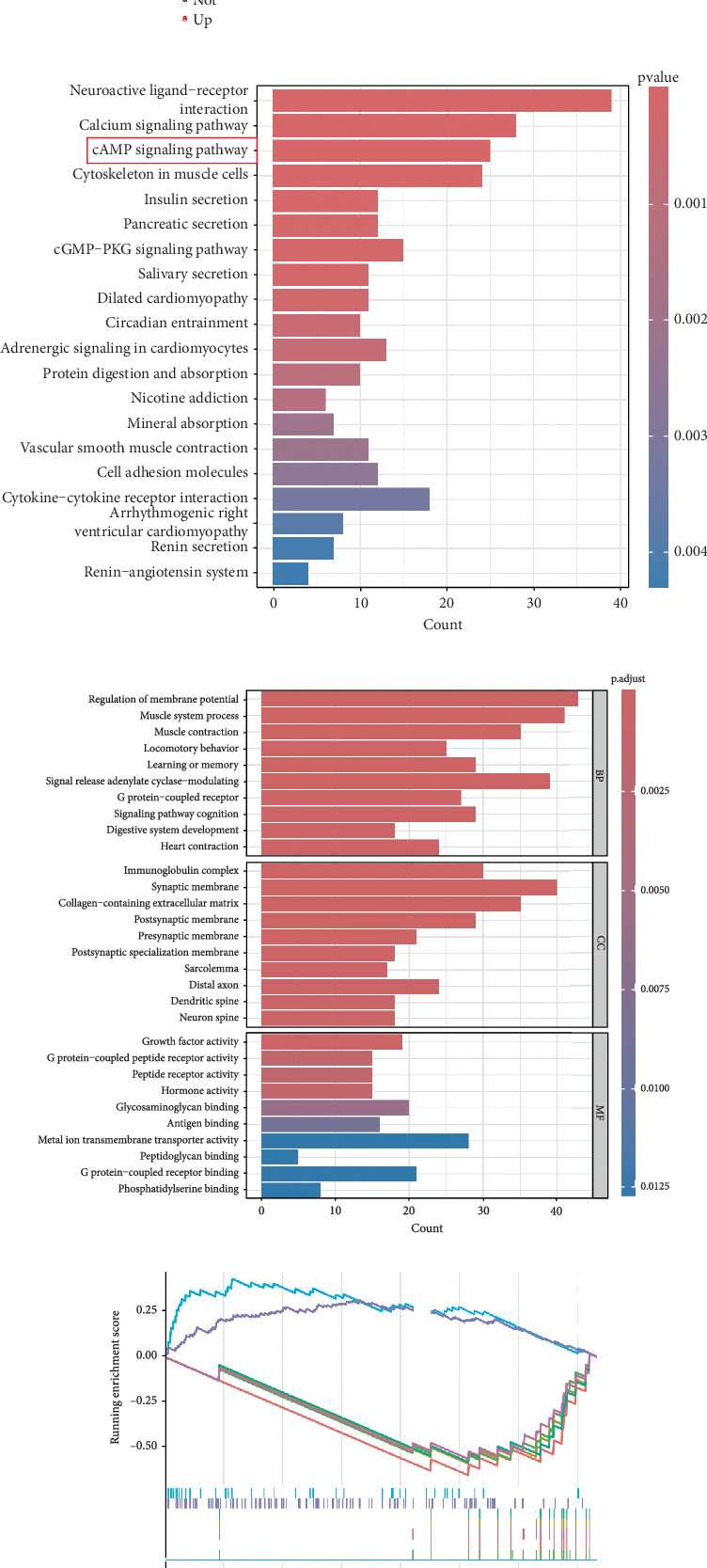
Differential expression genes and functional enrichment analysis of FENDRR. (a) Volcano plot of DEGs. (b) KEGG analysis results of FENDRR-related DEGs. (c) GO analysis results of FENDRR-related DEGs. (d) GSEA results of FENDRR-related DEGs.

This investigation was aimed at elucidating FENDRR's functional significance in COAD pathogenesis. To distinguish genes with varying expression levels between groups with high and low FENDRR expression, a volcano plot was constructed using thresholds of *p* < 0.05 and Log2 (fold change) > 1 or Log2 (fold change) < −1 applied to generate a volcano plot (Figure 3a). The red part of the plot corresponds to the upregulated genes, while the blue part corresponds to the downregulated genes. Subsequently, the *p* values were arranged in descending order, and the top 10 gene names of the up- and downregulated genes were annotated in the volcano plot. Additionally, functional enrichment assays were conducted to validate FENDRR's involvement in COAD. According to KEGG analysis, differentially expressed genes were predominantly associated with key pathways, including calcium and cAMP signaling (Figure 3b). The ten most statistically significant entries in BP, CC, and MF were selected based on their lowest *p* values (Figure 3c). The results suggested that the mechanism by which FENDRR affects COAD progression through mechanisms involving the extracellular matrix, growth factor activity, and G protein-coupled peptide receptor activity. These findings reveal that FENDRR plays a complex role in COAD through multiple biological processes. However, GO and KEGG, as conventional methods of differential analysis, may overlook certain genes with small expression differences but still have biological significance, thus failing to adequately consider the functional significance of gene regulatory networks as well as gene interactions. In order to overcome the limitations of the study, a more in-depth analysis of the role of FENDRR in COAD was performed using GSEA (Figure 3d). The GSEA results revealed that FENDRR is linked to biological processes such as muscle system regulation and cornification. Overall, this approach offered a more thorough exploration of FENDRR's functional mechanisms.

### 3.5. Correlation Between FENDRR Expression and Tumor Immune Microenvironment

**Figure 4:**
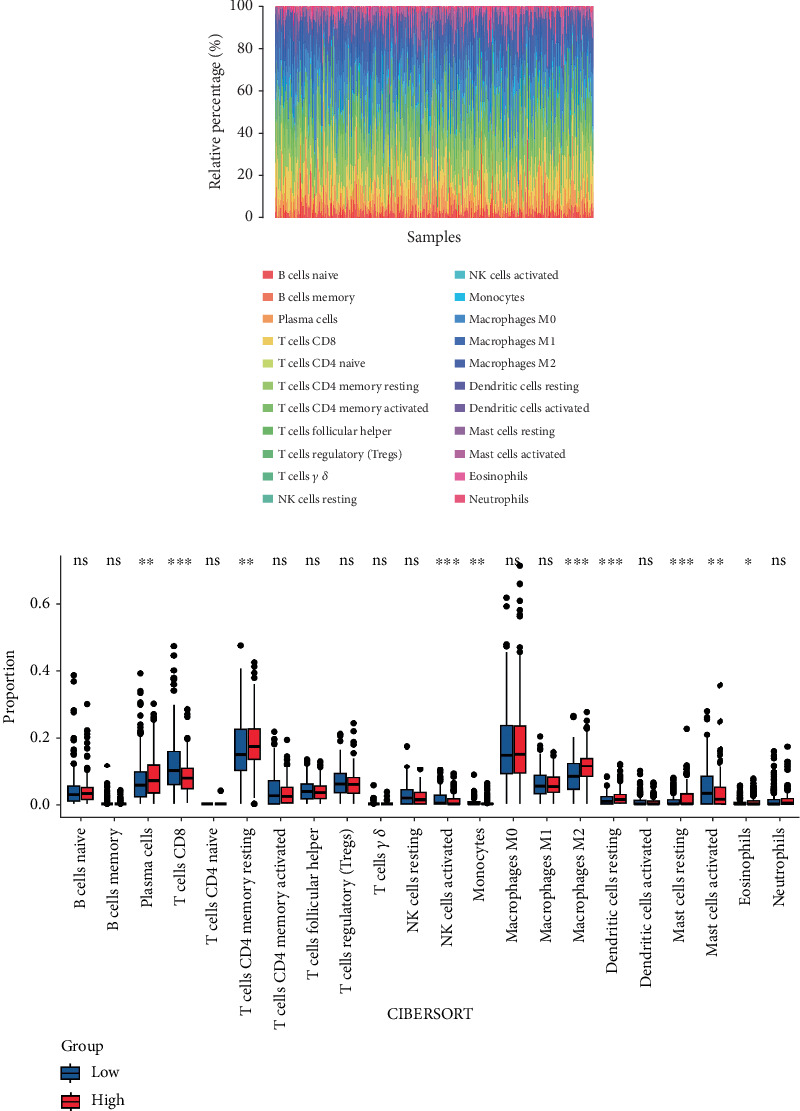
Tumor immune microenvironment analysis. (a) Proportion of various immune cell types across patient samples. (b) Differential immune cell infiltration between FENDRR high-expression and low-expression groups.

CIBERSORT analysis demonstrated distinct immune cell infiltration patterns in COAD patients based on FENDRR expression levels within TCGA dataset (Figure 4a,b). Compared to the low-expression cohort, the high-FENDRR group exhibited elevated proportions of plasma cells, resting memory CD4+ T cells, macrophage M2, resting dendritic cells, and eosinophils, alongside reduced CD8+ T cells, activated NK cells, monocytes, and resting mast cells (*p* < 0.05). No notable variations were detected in the remaining tumor-infiltrating immune cell populations. These observations imply that FENDRR may shape the tumor microenvironment, potentially modulating cancer progression through immune regulation.

### 3.6. Overexpression of FENDRR Suppresses the Proliferation of Colon Cancer Cells In Vitro

**Figure 5:**
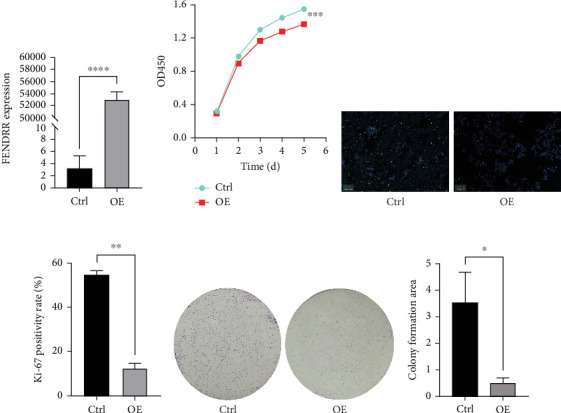
In vitro functional analysis of FENDRR in colon cancer cell proliferation. (a) qPCR validation of FENDRR overexpression transfection efficiency. (b) Cell viability assessed by CCK-8 assays. (c, d) Expression analysis of proliferation-related protein Ki-67. (e, f) Colony formation assays of the impact of FENDRR overexpression on the proliferation of colon cancer cells.

To explore the role of FENDRR in colon cancer development, we employed lentiviral transfection to achieve ectopic FENDRR overexpression. As demonstrated in Figure 5a, qPCR analysis confirmed the efficiency of the FENDRR transfection in HCT116 cells. Subsequent functional assays were conducted to assess FENDRR's impact on proliferation, including CCK-8 viability tests, Ki-67 IF staining, and clonogenic survival assays. The CCK-8 showed significantly slowed growth kinetics in FENDRR-overexpressing cells across all measured intervals (Figure 5b). IF analysis further indicated downregulation of the proliferation marker Ki-67 following FENDRR overexpression (Figure 5c,d). Additionally, colony formation assays revealed impaired colony-forming ability in FENDRR-transfected cells (Figure 5e,f). Consequently, these results collectively indicate that FENDRR overexpression inhibits colon cancer cell proliferation in vitro.

### 3.7. Overexpression of FENDRR Inhibits the Migration and Invasion of Colon Cancer Cells In Vitro

**Figure 6:**
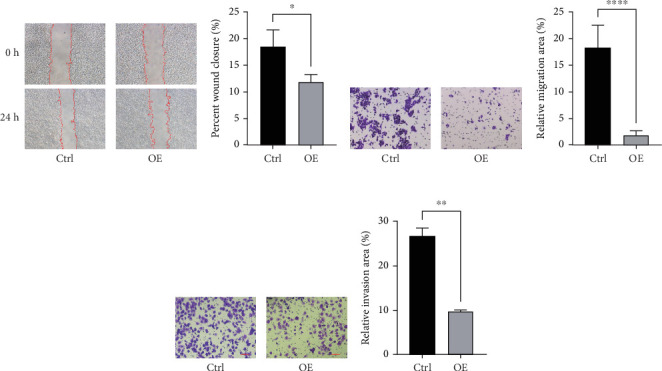
In vitro functional analysis of FENDRR in colon cancer cell migration and invasion. (a, b) Wound healing assays of the impact of FENDRR overexpression on the migration of colon cancer cells. (c–f) Transwell assay analysis of the impact of FENDRR overexpression on the migration and invasion of colon cancer cells (⁣^∗^*p* < 0.05, ⁣^∗∗^*p* < 0.01, and ⁣^∗∗∗^*p* < 0.001).

To assess FENDRR's influence on metastatic potential, we conducted complementary motility studies including wound healing assays and transwell assays (with or without Matrigel). The results of the wound healing assays and transwell assays (without Matrigel) revealed that the overexpression of FENDRR inhibited the migration ability of cells (Figures 6a, 6b, 6c, and 6d). Similarly, transwell assays (with Matrigel) demonstrated that the overexpression of FENDRR inhibited the invasive capacity of colon cancer cells (Figure 6e,f). Therefore, in in vitro experiments, FENDRR showed inhibitory effects on the migration and invasion ability of colon cancer cells.

### 3.8. Silencing Effect of FENDRR on the DUSP4/CREB/PRKACB Pathway

**Figure 7:**
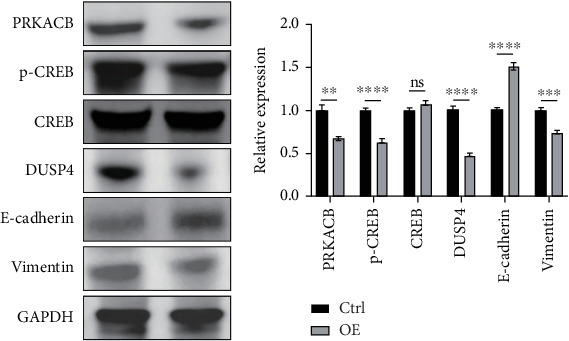
Western blot analysis of protein expression profiles. (a, b) Effect of FENDRR on DUSP4/CREB/PRKACB pathway proteins and epithelial–mesenchymal transition (EMT) pathway proteins (ns = p > 0.05, ⁣^∗∗^*p* < 0.01, ⁣^∗∗∗^*p* < 0.001, and ⁣^∗∗∗∗^*p* < 0.0001).

KEGG enrichment analysis indicated that the mechanism of FENDRR's involvement in colon cancer development might be associated with the cAMP signaling pathway, and the typical proteins of the cAMP signaling pathway are PKA and CREB. Differential expression analysis revealed a statistically significant inverse correlation between FENDRR expression and DUSP4 levels (*p* < 0.05). Concurrently, relevant studies have clearly reported the promoting effect of DUSP4/CREB/PRKACB on tumor progression. In light of the aforementioned findings, a hypothesis was formulated proposing that FENDRR exerts its inhibitory effect on the DUSP4/CREB/PRKACB pathway. To validate this hypothesis, we performed Western blot to quantify key components of the proposed signaling axis (DUSP4, CREB, p-CREB, and PRKACB). As demonstrated in Figure 7a,b, cells overexpressing FENDRR showed a marked decrease in DUSP4, p-CREB, and PRKACB levels compared to the control (*p* < 0.05), whereas CREB expression remained unchanged (*p* > 0.05). Aforementioned findings suggest that FENDRR inhibits the DUSP4/CREB/PRKACB pathway through the reduction of protein expression or the inhibition of protein phosphorylation.

### 3.9. Reversal Effect of FENDRR on the EMT Pathway

The in vitro experiment results revealed an association between FENDRR and the migratory and invasive potential of tumor cells, and EMT exerts a significant influence on the process of tumor migration and invasion. During EMT, epithelial cells experience a loss of cellular morphology, and a decline in cell adhesion, cytoskeletal remodeling, and cellular maneuvering increases contribute to tumor metastasis. Western blot analysis was performed to assess EMT-related protein expression. Figure 7a,b illustrates that FENDRR-overexpressing cells exhibited substantially lower levels of the mesenchymal marker vimentin compared to controls (*p* < 0.05), while E-cadherin, an epithelial marker, was significantly upregulated (*p* < 0.05). The above results indicated that FENDRR could reverse the development of EMT, thereby inhibiting colon cancer cell migration and invasion.

## 4. Discussion

CRC is the most typical malignant tumor worldwide. It is evident that certain lncRNAs modulate gene expression and are implicated in the development of CRC, functioning as either oncogenes or tumor suppressor genes. FENDRR, a specific type of lncRNA, has been shown to play a pivotal regulatory role in organ development, particularly in the context of heart formation and other organ development. It has been demonstrated that FENDRR exerts its regulatory influence on gene expression, thereby promoting normal tissue differentiation and organ development [32]. Recent studies on the function of FENDRR have predominantly focused on its role in cancer. The literature documents that FENDRR plays a crucial role in the progression of various malignant tumors, including colorectal, prostate, lung, gastric, bladder, and cervical cancers, consistently exhibiting tumor-suppressive effects [33–39]. Among them, in CRC, FENDRR was shown to correlate with the tumor microenvironment and immunogenicity [40, 41]. Our investigation revealed markedly decreased FENDRR expression in tumor specimens relative to adjacent normal tissues. Lower levels of FENDRR exhibited a strong correlation with advanced stage and poorer OS, thus establishing it as an independent protective factor. Consequently, further investigation is warranted into the biological activity of FENDRR on COAD tumor progression and the underlying mechanisms. Enrichment analysis demonstrated that FENDRR exerts an influence on intracellular signaling involving cAMP, calcium signaling pathway, and other such processes. In vitro experiments revealed that FENDRR effectively suppresses cancer cell growth, motility, and invasive capacity in COAD. These results strongly suggest FENDRR's therapeutic potential as both a treatment target and predictive indicator for disease outcomes. Existing research on colorectal malignancies has established that FENDRR significantly inhibits tumor cell proliferation, migration, invasion, and angiogenesis. The proteins involved in the underlying mechanisms include SOX4, ING4, miR-18a-5p, FBX8, and GSTP1 [42]. Additionally, the EMT process has been recognized as a critical pathway linking FENDRR to cancer spread, with supporting evidence from bladder carcinoma investigations [29].

In order to explore the new basis of FENDRR as a new target for COAD treatment, an analysis was conducted of the differential genes between high- and low-expression groups of FENDRR via the expression profiles from TCGA database. This analysis identified DUSP4, a gene that was significantly negatively correlated with FENDRR. A review of the literature revealed a close relationship between DUSP4 and the cAMP signaling pathway via the DUSP4/CREB/PRKACB axis. Intriguingly, the gene function enrichment analysis results also indicated a significant association between FENDRR and the cAMP signaling pathway. Consequently, the scientific hypothesis was formulated that FENDRR impacts the development of COAD by modulating the DUSP4/CREB/PRKACB axis. In order to test this hypothesis, FENDRR-overexpressing cell lines were constructed for WB experiments in order to verify the relationship between FENDRR and DUSP4, p-CREB, CREB, and PRKACB. In view of the findings of preceding studies, it was determined in the present study that FENDRR curtails persistent tumor growth by instigating the sinking of the DUSP4/CREB/PRKACB axis. In addition to this, a comprehensive investigation was conducted into the impact of FENDRR on EMT in colon cancer, with particular emphasis on its capacity to impede cell migration and invasion. The results demonstrated that FENDRR could reverse the occurrence of EMT and thus inhibit the migration and invasion of tumor cells.

It must be acknowledged that the present study is not without its limitations. Firstly, the validation of FENDRR expression through experiments involving 10 clinical specimens of COAD, although statistically noteworthy, constitutes a restricted sample size. Limited sample sizes heighten the risk of outliers and diminish statistical strength. Subsequent research should confirm these outcomes in larger, multicenter cohorts to guarantee their reliability. Secondly, we did not perform in vivo experiments in mice or advanced models, such as organoids, to validate the impact of FENDRR on the biological behavior of COAD, which will be a key focus of our next study.

## 5. Conclusion

In conclusion, our study provides new insights into COAD and further confirms the potential that FENDRR can be used as a new target for colon cancer treatment and a prognostic predictive biomarker, making it potentially clinically valuable for cancer screening, diagnosis, treatment evaluation, and prognostic follow-up.

## Data Availability

The data that support the findings of this study are available from the corresponding authors upon reasonable request.

## Supporting Information

Supporting Information Additional supporting information can be found online in the Supporting Information section. We have provided the supporting information for Table S1 and Table S2 mentioned in the Results section of the main text.
