# Supplementary material for: FENDRR Affects COAD Biological Behavior by Inhibiting the DUSP4/CREB/PRKACB Pathway
Source: Int J Genomics. 2025 Jul 1;2025:2765511. doi: 10.1155/ijog/2765511 (PMC12237554; doi:10.1155/ijog/2765511)
Supplement: Supporting Information — Additional supporting information can be found online in the Supporting Information section. We have provided the supporting information for Table S1 and Table S2 mentioned in the Results section of the main text. [file 2765511.f1.zip › Table S1.docx]

| Variables | High-COAD  (n = 200) | | | Low-COAD  (n = 193) | P | High-READ  (n = 70) | Low-READ  (n = 65) | P |
| --- | --- | --- | --- | --- | --- | --- | --- | --- |
| Age, n (%) |  | | |  | 0.804 |  |  | 0.111 |
| <60 | 54 (27) | | | 49 (25) |  | 17 (24) | 25 (38) |  |
| ≥60 | 146 (73) | | | 144 (75) |  | 53 (76) | 40 (62) |  |
| Gender, n (%) |  | | |  | 0.566 |  |  | 0.064 |
| Female | 98 (49) | | | 88 (46) |  | 38 (54) | 24 (37) |  |
| Male | 102 (51) | | | 105 (54) |  | 32 (46) | 41 (63) |  |
| TNM Stage, n (%) |  | | |  | 0.757 |  |  | 0.964 |
| I-II | 113 (56) | | | 113 (59) |  | 39 (56) | 35 (54) |  |
| III-IV | 87 (44) | | | 80 (41) |  | 31 (44) | 30 (46) |  |
| T Stage, n (%) |  | | |  | 0.008 |  |  | 1 |
| T1-T2 | 49 (24) | | | 26 (13) |  | 17 (24) | 15 (23) |  |
| T3-T4 | 151 (76) | | | 167 (87) |  | 53 (76) | 50 (77) |  |
| N Stage, n (%) |  | | |  | 0.595 |  |  | 0.832 |
| N0 | 116 (58) | | | 118 (61) |  | 40 (57) | 35 (54) |  |
| N1-N2 | 84 (42) | | | 75 (39) |  | 30 (43) | 30 (46) |  |
| M Stage, n (%) |  | | |  | 0.572 |  |  | 1 |
| M0 | 170 (85) | | | 159 (82) |  | 59 (84) | 55 (85) |  |
| M1 | 30 (15) | | | 34 (18) |  | 11 (16) | 10 (15) |  |
|  |  |  |  |  |  |  |  |  |
|  |  |  |  |  |  |  |  |  |
|  |  |  |  |  |  |  |  |  |
|  |  |  |  |  |  |  |  |  |
|  |  |  |  |  |  |  |  |  |
|  |  |  |  |  |  |  |  |  |
|  |  |  |  |  |  |  |  |  |
|  |  |  |  |  |  |  |  |  |
|  |  |  |  |  |  |  |  |  |
|  |  |  |  |  |  |  |  |  |
|  |  |  |  |  |  |  |  |  |
|  |  |  |  |  |  |  |  |  |
|  |  |  |  |  |  |  |  |  |
|  |  |  |  |  |  |  |  |  |
|  |  |  |  |  |  |  |  |  |
|  |  |  |  |  |  |  |  |  |
|  |  |  |  |  |  |  |  |  |
|  |  |  |  |  |  |  |  |  |
|  |  |  |  |  |  |  |  |  |
